# Features and Components Preferred by Adolescents in Smartphone Apps for the Promotion of Physical Activity: Focus Group Study

**DOI:** 10.2196/33972

**Published:** 2022-06-09

**Authors:** Alex Domin, Yacine Ouzzahra, Claus Vögele

**Affiliations:** 1 Research Group for Self-Regulation and Health Department of Behavioural and Cognitive Sciences University of Luxembourg Esch-sur-Alzette Luxembourg; 2 Research Support Department University of Luxembourg Esch-sur-Alzette Luxembourg

**Keywords:** mHealth, physical activity, mobile phone, health, qualitative research, focus groups, smartphone apps, behavior change, mobile health, adolescents

## Abstract

**Background:**

There is solid evidence that lack of physical activity (PA) is a risk factor for chronic diseases. Sufficient levels of PA in childhood and adolescence are particularly important, as they can set the standards for PA levels in adulthood. The latest reports show that only a small percentage of adolescents reach the recommended levels of PA in European Union countries at the age of 15 years. In view of the scale of the problem, it is crucial to develop interventions that promote and support PA in adolescents. Considering their low implementation costs and ubiquitous presence, smartphone apps could be advantageous as a part of PA interventions.

**Objective:**

This study aimed at investigating the attitudes and preferences of adolescents aged 16-18 years toward various PA app features and components that could (1) make the app more attractive for them and consequently (2) increase their interest and engagement with the app.

**Methods:**

Two separate focus group discussions were conducted in 2 groups of adolescents (n=4 each) aged 16-18 years. Focus groups were carried out online via video conference. The discussions were conducted using a semistructured interview. Participants (n=8; 4 males and 4 females) had a mean age of 17.25 years (SD 0.82 years). Transcripts were analyzed following the approach by Krueger and Casey, that is, categorizing participants’ answers and comments according to the questions and themes from the focus group schedule.

**Results:**

Features, such as “goal setting and planning,” “coaching and training programs,” “activity tracking,” “feedback,” and “location tracking” were appraised as attractive, motivating, and interesting. An “automatic activity recognition” feature was perceived as useful only under the condition that its precision was high. The “reminders” component was also deemed as useful only if a range of conditions was fulfilled (timeliness, opportunity for customization, etc). The features “mood and sleep tracking,” “sharing workout results via social networks,” “digital avatar and coach,” and “rewards” were generally perceived negatively and considered as useless and not motivating. In general, participants preferred features with an easy-to-navigate interface and a clear, simplistic, and straightforward layout with a modern design. Customization and personalization qualities were highly appreciated throughout an app, together with data precision.

**Conclusions:**

This study contributes to the understanding of the features and components preferred by adolescents in apps promoting PA. Such apps should provide users with precise data, and have a simplistic modern design and a straightforward easy-to-use interface. Apps should be personalized and customizable. Desired features to be included in an app are goal setting and planning, feedback, coaching and training programs, and activity tracking. The features should involve high levels of data precision and timely delivery while taking into consideration the real-life context.

## Introduction

Physical activity (PA) has been shown to be beneficial for both mental and physical health, while a lack thereof is known to be a risk factor for chronic and cardiovascular diseases [1]. Sufficient levels of PA in childhood and adolescence are particularly important, as it can set the standards for PA levels in adulthood [2]. The latest reports show that less than 20% of girls and 25% of boys reach the recommended levels of PA in European Union countries at the age of 15 years [1,3]. In view of the scale of the problem, it is crucial to develop interventions that promote and support PA in adolescents globally. Adolescents aged 16-18 years deserve special attention, as they display the lowest absolute PA levels among adolescents aged 5-19 years and are considered an at-risk group [4,5].

Smartphones have become ubiquitous devices among the young population over the last few years. In 2019, an estimated 94% of European young people accessed the internet on a daily basis, and 92% used mobile phones to access the internet away from home and work, according to Eurostat [6]. Considering their low implementation costs and pervasive presence, smartphones could be advantageous as a part of PA interventions, while also benefiting from multiple built-in sensors (eg, accelerometer, pedometer, GPS sensor, camera, and microphone) [7].

Smartphone apps are available through popular digital distribution services or app markets (eg, Apple App Store, Google Play Store, and Windows Phone Store). A recent review of apps aiming at improving diet, increasing PA, and reducing sedentary behavior in children and adolescents suggested that (1) there are fewer apps developed for adolescents than for adults; (2) the quality of apps is moderate, scoring the lowest for information quality and demonstrating a lack of theory-based (behavior change techniques and theories) and evidence-based (PA guidelines) approaches, which is congruent with reviews of PA apps for adults; (3) more formative research is needed to better understand the factors that improve adolescents’ engagement and app quality [8]. Despite the increased interest in this field, there is a paucity of studies exploring and developing smartphone apps that promote and support PA in adolescents. A recent scoping review analyzing a range of evidence (both quantitative and qualitative) available on smartphone-based mobile health (mHealth) PA interventions and looking into the development and evaluation trajectory of smartphone-based mHealth PA interventions identified a lack of qualitative and quantitative studies exploring adolescents’ views and experiences of apps promoting PA [9].

Existing qualitative studies exploring smartphone use for health purposes mainly focused on adult populations in several contexts, including PA [10-13], health behavior change [14,15], health and fitness [16], and well-being [17]. Sample sizes in these studies vary, and the methods of data collection range from focus groups to online surveys, interviews, and “think aloud” methods. For example, Rabin and Bock [10] used a combination of a survey and a semistructured interview to collect feedback on 3 PA apps that can guide the development of theory-based and empirically based apps incorporating preferences of adults. Ehlers and Huberty [11] used an online survey to identify theory-based behavioral and technological features preferred by middle-aged women. Middelweerd et al [12] used a series of focus group discussions to explore students’ preferences regarding a PA app. In their study, 30 participants aged between 18 and 25 years used the Nexercise app for 3 weeks and subsequently participated in a focus group discussion. Finally, Baretta et al [13] implemented a combination of a “think-aloud” methodology and in-depth interview techniques to examine the features of apps, such as “Runtastic Running & Fitness Tracker,” “Endomondo - Running & Walking,” and “Runkeeper - GPS Track Run Walk,” which are important for users’ engagement during the first exposure and after 2 weeks of using 1 of the 3 commercial apps.

In order to address these gaps in the literature, 2 separate focus group discussions were conducted in 2 groups of adolescents (n=4 each) aged 16-18 years, to investigate their experiences, attitudes, and preferences toward various PA app features and technologies that could (1) make the app more attractive for them and consequently (2) increase their interest and engagement with the app. This formative study was conducted to better understand the factors that improve adolescent engagement and app quality, and to ultimately inform the development of a mobile app focused on promoting PA among adolescents. The following research question was addressed: what features and components are preferred by adolescents (aged 16-18 years) in apps promoting PA?

## Methods

### Recruitment

As we intended to target a sample of adolescents aged 16-18 years, local schools were chosen as a recruitment location. As a consequence of lockdown measures because of the COVID-19 pandemic, it was difficult to recruit participants directly from schools, so we eventually used social networks as a recruitment platform. Students, who were interested and owned a smartphone, were initially asked to complete a questionnaire assessing their level of PA (Physical Activity Questionnaire for adolescents [PAQ-A]) [18,19], demographics, and experience with PA apps. Participants and their parents were asked to sign an informed consent form before taking part in the discussion session. An effort was made to include participants with both high and low levels of PA, as individual PA profiles may affect the preference of certain features of PA apps [12]. The PAQ-A questionnaire score ranges from 1 (low PA) to 5 (high PA). After completing the questionnaire, participants were divided into the following 2 focus groups: a group with participants who had a PAQ-A score below 3 (low level of weekly PA), and a group with participants who had a PAQ-A score of 3 or above (moderate to high level of weekly PA). To ensure representativeness of the focus groups, additional attention was paid to gender balance in both groups.

### Design

The design was guided by recommendations on the appropriate conduct of focus group discussions provided by Breen [20], and Krueger and Casey [21]. Both focus groups were carried out in Luxembourg online, using the videoconferencing software Skype for Business 2016. The moderator had previous experience of conducting qualitative research using interviewing techniques. Discussions were conducted using a semistructured interview guide. The moderator anticipated 90 minutes for each discussion; however, sessions could be prolonged, if needed. Both focus group discussions were audio and video recorded and transcribed verbatim, and the data were pseudonymized. A small incentive (€20 [US $21] voucher) was sent to participants after their participation in the discussion session.

### Ethics Approval

The study was approved by the Ethics Review Panel of the University of Luxembourg (ERP 19-046A2 MAPA).

### Participants

Eligible participants (n=10) were students between 16 and 18 years of age. Due to dropout related to technical difficulties, only 8 participants took part in the focus group discussions. Participants were required to own a smartphone with internet access and to have some experience with PA apps prior to the session to ensure a meaningful focus group discussion. The sessions were conducted in English; therefore, all participants were required to have a sufficient command of the English language. Eligible participants were required to be healthy and to have no contraindications for PA participation.

### Procedure

The 2 focus groups were held on separate days. Prior to the focus group discussions, signed consent forms were sent to the moderator. The moderator tested participants’ language comprehension before commencing with the focus group discussion, and to avoid language barriers, the moderator used plain English and rephrased questions when needed. Then, during the online focus group session, the moderator welcomed participants and proceeded with the general overview of the topic. Further, the moderator ensured that participants were aware of the purpose of the study and its procedures, stated the ground rules for the focus group discussion, and underlined that the ensuing discussion was audio and video recorded for research purposes, assuring confidentiality and anonymity of transcriptions [20].

The focus group discussion started with a sequence of questions previously described by Dennison (Multimedia Appendix 1) [14]. Participants were initially asked to describe how often they use their smartphones, for which purposes, and which apps were used the most. In the next step, participants answered questions about their personal experience of smartphone apps for PA. To prompt a further discussion, the moderator used trigger materials that were explained and distributed among the participants (Multimedia Appendix 1). These materials included graphic examples of the most popular components present in both commercial and research-grade apps aimed at promoting PA. The list of app components presented to participants is shown in Textbox 1. The moderator offered a summary of key questions and sought confirmation from participants.

After the interviewer presented each app component, participants were asked to comment on their thoughts and feelings in terms of perceived usefulness and relevance. Next, participants were asked to write their own “ideal” rewards that would motivate them to be more physically active in the chat window. At the final stage of the discussion, participants were asked questions about subsequent app development (“Questions specific for the MAPA app development trial” section in Multimedia Appendix 1). The analysis of this section was not included in this study. Although the focus group discussions were using a semistructured interview approach, sessions were conducted as “guided conversations,” enabling the discussion to flow into unexpected directions [15]. At the end of the session, participants received the incentive as a token of gratitude.

**App components presented to the participants.**

**App components**

1. Goal setting and planning
2. Coaching and training programs
3. Activity tracking
4. Mood and sleep tracking
5. Feedback
6. Sharing workout results via social networks
7. Social support and comparison (in-app social profile and challenges)
8. Location tracking
9. Automatic activity recognition
10. Digital avatar and coach
11. Rewards (virtual)
12. Reminders

### Data Collection, Management, and Analysis

Both focus group discussions were video and audio recorded, and transcribed verbatim. A pseudonym was created for every participant to ensure anonymity. The transcripts were analyzed using the focus group discussion analysis methodology described by Krueger and Casey [21], which consist of categorizing participants’ answers and comments according to the questions and themes from the focus group schedule using a word processor and consequently writing a descriptive summary for answers to each question or theme. A student assistant was involved in the transcription, coding, and categorization process. The fragments extracted from the transcripts were split according to the respective focus group discussion. This was done to identify differences between the 2 groups defined by their PA level. The data were later combined for further analysis [12].

## Results

### Overview

Among 10 eligible participants, 8 took part in focus group discussions (Table 1). Among the 8 participants, 2 were aged 16 years, 2 were aged 17 years, and 4 were aged 18 years (mean age 17.25 years, SD 0.82 years). To ensure gender balance, an equal number of males (n=4) and females (n=4) were enrolled. All participants were living in Luxembourg and studying in local schools (upper secondary education). All participants stated that they had no health issues or any other limitations preventing them from participating in any type of PA. Only 4 participants reported moderate to high levels of weekly PA, as assessed by the PAQ-A questionnaire. Every participant owned a smartphone and had experience of using at least one fitness app, mostly Garmin TrainingPeaks, Strava, Nike Training Club, or Adidas Runtastic, tracking mainly such activities as running, cycling, (gym) workouts, and swimming. Participants provided comments on various app components, which were further summarized as key themes.

**Table 1:** Participants’ characteristics.

Focus group number	Age (years)	Gender	Grade	PAQ-A^a^ score	Performed sports	Physical activity apps used
1	16	Female	3	2.75	Cycling and running	Garmin TrainingPeaks
1	18	Female	1	2.75	Running	Garmin TrainingPeaks and Strava
1	18	Female	1	2.6	Cycling, running, and rugby	Garmin TrainingPeaks, Strava, and Nike Training Club
1	16	Male	3	2.8	Cycling, running, soccer, and gym workouts	Garmin TrainingPeaks and Strava
2	18	Male	1	4.35	Cycling, running, soccer, and basketball	Adidas Runtastic
2	17	Male	2	3.5	Cycling, running, soccer, and basketball	Garmin TrainingPeaks
2	18	Female	1	3.68	Cycling, running, swimming, and volleyball	Adidas Runtastic, Garmin TrainingPeaks, and Nike Training Club
2	17	Male	2	3	Cycling, running, soccer, and skateboarding	Garmin TrainingPeaks

^a^PAQ-A: Physical Activity Questionnaire for adolescents.

### Ubiquitous Themes

Throughout the majority of discussed topics in both focus groups, certain comments indirectly related to the initial questions resurfaced on multiple occasions. These comments were united into “ubiquitous themes” related to app features. These themes were centered around design, customization and personalization, and data precision.

#### Design

Participants preferred a clear and simplistic app layout with an easy-to-navigate interface. Overall, apps were appraised while having modern looks and appealing colors (Adidas Runtastic and Garmin TrainingPeaks). Participants also preferred information to be displayed in a logical and straightforward manner. Any irrelevant or overcomplicated data were disliked, together with a scattered or complex layout.

#### Customization and Personalization

For an app to be appealing, it had to combine such qualities as flexibility and diversity. While customization refers to adjustments done by users, personalization refers to adjustments done by an app or a platform. Specifically, the majority of the features were perceived as appealing when the content was personalized and a user was able to customize it toward her/his preferences (hiding or unhiding various features, changing colors, etc). A user should be able to turn on/off different features (eg, location tracking), and the content (eg, proposed workouts) should be diverse and customizable.

#### Data Precision

Various app features were only perceived as useful if data provided in the feedback tab were precise and accurate; otherwise, they were perceived as useless and ineffectual.

### Differences Between Focus Groups

The analysis showed agreement concerning general themes between both groups. The only difference between group 1 (participants with low PA levels) and group 2 (participants with moderate and high PA levels) concerned the perception of the “social support and comparison” feature (in-app social profile and challenges). This feature involves exposing user’s activity and achievements within an app’s social ecosystem. While group 1 appreciated this feature and perceived it as motivating and fun, group 2 mainly disliked it (accounting for the mental pressure this feature puts on participants).

So, during the quarantine we had like a running challenge where everyone who has this app could participate and the one with the most kilometers won. So, I really like that. It kind of motivated me to run because I didn't want to be last.

Group 1 participant, female

I don't like it. I went running with a lot of people who actually have this fitness app that can really track other people that you follow. And they always ask me if I wanted to do it but I didn’t, I just don't like it because I think that it puts a lot of pressure on you to be better than them and I want to run because I do it for myself and not for others so...

Group 2 participant, female

### General Themes

#### Goal Setting and Planning

This feature was generally perceived as useful and motivating (especially for those who do not train with a real-life coach). In general, participants enjoyed having the possibility to plan activities and have a clear outline of the activity agenda. They not only favored the possibility of choosing the preselected activities, but also enjoyed including their own activities as planning options.

#### Coaching and Training Programs

In general, this feature was perceived positively and appraised as especially useful for beginners who do not have a real-life coach. Participants outlined the motivational value of a coach (real-life or online, eg, a familiar athlete), as well as the importance of flexibility and variability in online training programs. They enjoyed a more personalized interface and the ability to customize their training plan. Furthermore, they enjoyed reviewing the workout time frame and the training sample videos. Concurrently, the participants appraised some of the information as not useful (eg, calories burned during certain workouts). They outlined the importance of indicating the difficulty level and the equipment required for workout sessions.

I also like that the app has different options… And I also think the calories stuff is useless because it changes from person to person. So, it could be better if they told you whether it's a hard workout or just an easy one.

Group 2 participant, male

#### Activity Tracking

Participants appreciated the activity tracking feature, specifically for such components as location, pace, duration, and distance tracking. Moreover, they proposed to integrate the audio player with the tracking interface (in order to simultaneously track the activity and listen to music) and underlined that an app has to request users for permission to track their location.

I like all of them because they all track the location and duration of the run, the distance. And there's nothing too, too complicated about it. You can just go with the map or listen to music also. I think it's yeah, it's a good thing to do.

Group 1 participant, female

#### Mood and Sleep Tracking

Overall, participants did not perceive this feature as important and consequently gave mixed feedback. In general, they liked the idea of having data about sleep duration and mood assessment, yet only if it was precise. Participants shared their past (mostly negative) experiences and underlined that when such information is inaccurate, it is useless and they would not benefit from it.

I used lots of things, in the beginning when I had my smart watch for the first time. And actually, noticed that it isn't accurate. So, it said, I go to sleep at 9 o'clock when I actually go to sleep at 11 clock, so...

Group 2 participant, female

#### Feedback

This feature was perceived very positively, but only on the condition of appropriate design (clear, simplistic, customizable, and only relevant information). Participants enjoyed the opportunity to review their monthly, weekly, or daily achievements.

I think that's probably one of the most important parts of an app. Because you can see all your data. Yeah so it needs to be very clean. Yeah. Not too much going on.

Group 2 participant, male

I like the color (Garmin app). I like that you're running once a day and activity is in orange. So, it jumps out. So, it's the first thing you see. You have the most important information next to it, so... I don't really like the other one [Fitbit app]. It's just too much. I don't need to see how many floors I climbed that day. It's just a bit useless.

Group 1 participant, female

#### Sharing Workout Results via Social Networks

The majority of participants disliked this feature and would not want to share their activity results via social networks. They suggested this feature to be an optional component for other people who are active on social media but not for everyone.

#### Location Tracking

This feature was appraised positively and was useful for tracking various PAs. It was used for navigation purposes or for discovering and exploring new areas near home and during vacation. However, its function to run in the background throughout the day was not appreciated. Participants outlined that there has to be a clear way to turn the tracking feature off, and an app has to notify its users about tracking their location.

#### Automatic Activity Recognition

In general, participants found this feature useful only if the precision level was high; otherwise, it was considered unnecessary and useless according to participants past experiences.

I don't think it's very like precise or anything. But I don't really mind it. I think it can be useful if it's like precise. I mean I don't really need to know how much or how many minutes I walked. But yeah I just think it might be like fun to like look what they recognize as an exercise...

Group 1 participant, female

#### Digital Avatar and Coach

In essence, this feature was perceived negatively and assessed as not motivating, useless, and unprofessional (associated rather with a game than a PA app). It was regarded to be useful for other people that perform home workouts. It was proposed to have this feature as an optional component.

#### Rewards (Virtual)

This feature was generally disliked and described as childish, not motivating, and not evoking feelings of pride. The monetization did not make sense to participants, and they disliked the shift in focus from internal to external motivation. Participants recommended it as an optional feature for beginners. The ideal rewards suggested were items related to preferred activities or workouts (eg, equipment, clothes, in-app upgrades, and a possibility to unlock premium features).

#### Reminders

This feature received mixed feedback and was deemed as a useful feature only in particular cases (eg, for beginners or other people not training with a real-life coach and in combination with an already established training program). In addition, it was perceived as motivating only if it could be customized, could be turned off when needed, and would appear only when relevant (eg, not during lessons or after the training).

## Discussion

### Principal Findings

This exploratory study aimed at investigating the preferences and attitudes of adolescents (aged 16-18 years) toward various PA app features and technologies that could potentially make an app more attractive and consequently increase interest and engagement.

Features, such as “goal setting and planning,” “coaching and training programs,” “activity tracking,” “feedback,” and “location tracking,” were preferred by focus group participants, and were appraised as attractive, motivating, and interesting. The “automatic activity recognition” feature was perceived as useful only under the condition that its precision is high. The “reminders” component was also deemed as useful only if a range of conditions was fulfilled (timeliness, opportunity for customization, etc). The features “mood and sleep tracking,” “sharing workout results via social networks,” “digital avatar and coach,” and “rewards” were generally perceived negatively and were considered as useless and not motivating. In general, participants preferred when features had an easy-to-navigate interface and had a clear, simplistic, and straightforward layout with modern design. Customization and personalization qualities were highly appreciated throughout an app, together with data precision.

While the comparison of focus group participants with low PA levels and those with moderate to high PA levels showed agreement in the majority of app features, the groups differed in their preference for the “social support and comparison” component in that the former liked it better than the latter. This difference suggests that “social support and comparison” may not be a primary feature in PA apps for adolescents.

These findings support previous research conducted with adults, with some exceptions. While in line with the work of Rabin and Bock [10] confirming user preferences toward PA app features, such as user friendly interface, background music integration, goal setting, and tracking progress toward PA goals, our findings differ from the results reported by Ehlers and Huberty [11], who found that the most preferred technological features concerned components that enhance playfulness, competition with peers, and interaction in the app. The current findings are in line with most of the results reported by Middelweerd et al [12] in that users preferred a simple layout, the ability to tailor an app’s interface according to their needs, the tracking of PA using GPS, coaching features, tailored goals, and feedback. In contrast to these results, however, our participants had mixed thoughts concerning such features as competition with friends and a reward feature in the PA app. In addition, our findings are generally in line with the findings of Baretta et al [13], where features, such as simplicity, self-regulation skills support, and context tailoring, were perceived as important for users’ continuous engagement. Finally, when compared with the very few studies involving adolescents, our findings are in line with those of Lubans et al [22] and Seah and Koh [23], where features, such as goal setting, feedback, and activity tracking, were perceived as motivating by users.

### Implications for Future Interventions

As this study was conducted exclusively with a Luxembourgish sample, conclusions must be drawn with caution as the results cannot be generalized to other populations. In addition, and subsequent to this qualitative approach, future studies should use a quantitative design involving a sufficiently powered sample of adolescents. Nevertheless, even at this preliminary stage, the results point toward the importance of a number of features concerning PA apps for adolescents. A preliminary list of recommended app features that researchers and developers may want to take into consideration when developing an app promoting and supporting PA in adolescents is presented in Textbox 2.

**Preliminary list of recommended app features.**

**App features**

1. Design: An up-to-date easy-to-navigate interface with a clear, simplistic, and straightforward layout is required. Features, such as customization and personalization, are appreciated, yet without overcomplicating the app interface.
2. Data: Information provided to users should be timely and precise. Auxiliary features indirectly linked to physical activity (PA) (eg, sleep duration and quality) may only be included when supported by precise data; otherwise, they may be excluded.
3. Goal setting and planning: It may be recommended for inclusion. This feature should provide the possibility to plan activities (generic activity suggestions should be proposed, yet should also be customizable) and have a clear outline of the activity agenda.
4. Feedback (on previously performed PA): It may be recommended for inclusion while providing the possibility for review of monthly, weekly, and daily achievements.
5. Coaching and training programs: It may be recommended for inclusion while assuring customization and variability of online training programs. The proposed programs may be supported by training sample videos and information about the required equipment.
6. Activity tracking: It may be recommended for inclusion. Information should be provided about location, pace, duration, and distance of the exercise (when possible). Moreover, an audio player integrated with the tracking interface (in order to simultaneously track the activity and listen to music) may be advantageous. It is important, however, to underline that users should be able to turn off this feature. In addition, the app should notify the user about tracking details and ask for permission from the user for location tracking.
7. Location tracking (while not exercising): It may be recommended for inclusion while discovering and exploring new exercise areas near the home or during vacation, but not for tracking the location in the background throughout the day. Similar to activity tracking, the user should be able to turn off this feature, and the app should notify the user about tracking details and ask for permission from the user for location tracking.
8. Automatic activity recognition: It may only be included if the app can provide precise results.
9. Mood and sleep tracking: It may only be included if the app can provide precise data.
10. Sharing workout results via social networks: It may only be included as an optional component.
11. Social support and comparison (in-app social profile and challenges): It may only be included as an optional component.
12. Digital avatar and coach: It may only be included as an optional component.
13. Rewards: It may only be included as an optional component for beginners.
14. Reminders: It may only be included if this feature is timely, relevant to the current context, and can be customized and turned off when needed.

### Strengths and Limitations

To the best of our knowledge, this is the first qualitative study exploring adolescents’ views on PA app features. These results should be replicated in future studies using quantitative designs and systematically investigating potential gender effects. The current results suggest several features of smartphone-based PA interventions for teenagers that should be considered by developers and researchers.

There are several limitations in this study. First, we did not provide participants with a specific mobile platform (app), rather participants reviewed screenshots from different PA apps. It could be argued that participants’ assessments of app features may have differed when interacting with a functional app rather than viewing noninteractive screenshots. This should be replicated in future studies assessing participants’ views of specific apps during use. Second, the current sample consisted of individuals who at some point were members of a sports club (most often specialized in running), and this may have affected the results. Moreover, as the sample was quite small, future researchers should confirm the findings using more representative samples, achieving better data saturation, and including a sufficient number of boys and girls to systematically investigate any gender effects. The findings of this study, therefore, cannot be generalized beyond similar populations because of its qualitative explorative characteristic.

Taking into account these limitations, the findings of this study provide the first evidence of teenagers’ views on the features of PA promotion apps. It is hoped that this may stimulate future studies on larger and more representative samples, thereby providing conclusive evidence for developing effective PA promotion apps.

### Conclusions

This study contributes to the understanding of the features and components preferred by adolescents in apps promoting PA. Such apps should provide users with precise data, and have a simplistic modern design and a straightforward easy-to-use interface. Apps should be personalized and customizable, than is, have the ability to be tailored toward users’ needs and wishes. Desired features to be included in an app are goal setting and planning, feedback, coaching and training programs, and activity tracking. The features should involve high levels of data precision and timely delivery while taking into consideration the real-life context. This study provides initial information for both researchers and app designers working on the development of effective smartphone-based PA promotion interventions. Future quantitative studies should explore which app features could potentially increase motivation and improve long-term engagement of app users.

## Appendix

Multimedia Appendix 1 Focus group schedule and trigger materials.

## Abbreviations

PA: physical activity
PAQ-A: Physical Activity Questionnaire for adolescents
