# Supplementary material for: Features and Components Preferred by Adolescents in Smartphone Apps for the Promotion of Physical Activity: Focus Group Study
Source: JMIR Hum Factors. 2022 Jun 9;9(2):e33972. doi: 10.2196/33972 (PMC9227785; doi:10.2196/33972)
Supplement: Multimedia Appendix 1 [file humanfactors_v9i2e33972_app1.pdf]

Multimedia Appendix 1. Focus group schedule and trigger materials.

FOCUS GROUP SCHEDULE

The discussion will begin with questions around participants' personal experiences of smartphone apps for PA (Dennison et al., 2013):

1. Can we start by asking about whether you have had any experience of using your mobile phones for anything to do with PA?
2. Can you tell us about anything you have found useful to support making changes towards more active behavior (e.g. sitting less, moving more, doing sports, etc.)?
3. What sort of things (features) make you want to use it/stop using PA-related app?
4. When do you usually use the PA apps and for which kinds of activities? How often do you use them? Do you use the app for a long time or do you abandon it quickly (in several weeks/months)?

Participants are further shown **trigger materials** (see below) and after each section are asked to report their thoughts and feelings about the presented features of PA apps, including their perceived usefulness, relevance, and concerns (Dennison et al., 2013).

- What do you think/how do you feel about these?
- Is there anything about this you think would be useful for you?
- What about any problems or concerns you can see with this?

1. After discussing "reminders" component participants are then asked to comment the best time for reminders type and number of reminders. Participants are then asked to comment on their availability during the day (number of times, time and period), when they could follow app suggestions and perform PA).
2. After discussing "digital coach/avatar" component, participants are asked to comment on preferred digital assistant avatar; then on colors of the app interface and fonts.
3. After discussing "rewards" component, participants are asked to comment on their preferred reward (in the chat) and orally.
4. After discussing "reminders" component, each participant is asked to write an example of "ideal" notification in the chat that would motivate them to perform PA.

Questions specific for the MAPA app development trial:

1. Participants are then asked about their preferences for the type of PA, and asked on how do they decide on a performing a certain type of PA when they do not know what PA to engage into.
2. Participants are asked to share their typical schedule during the week (wake up and bed time) and during the weekend (wake up and bed time).
3. Participants are be asked to present their views on sharing data with others, including peers and private companies (e.g. Fitbit).

TRIGGER MATERIALS

Goal setting and planning

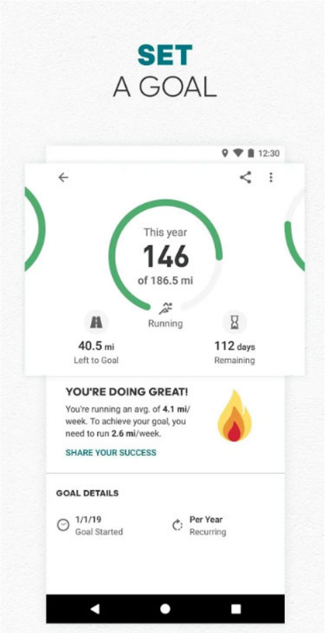

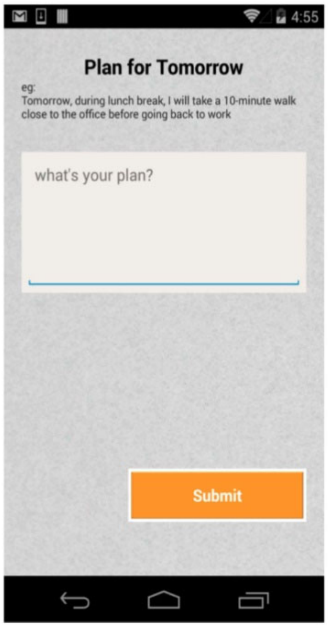

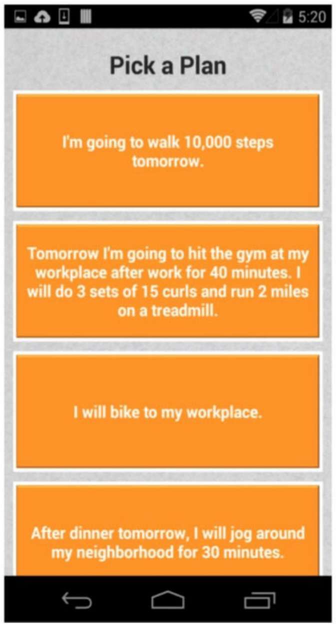

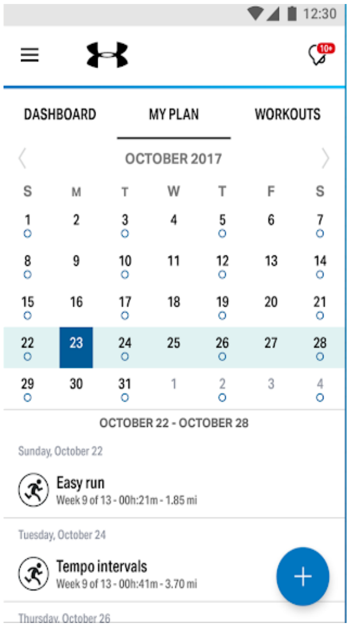

Coaching and training programs

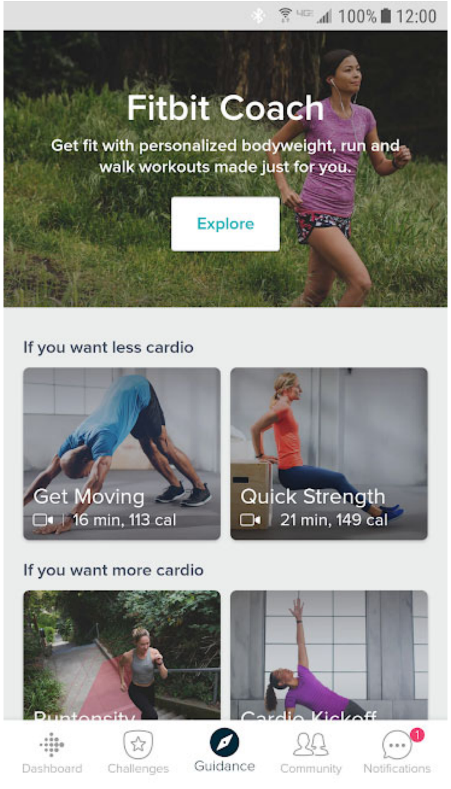

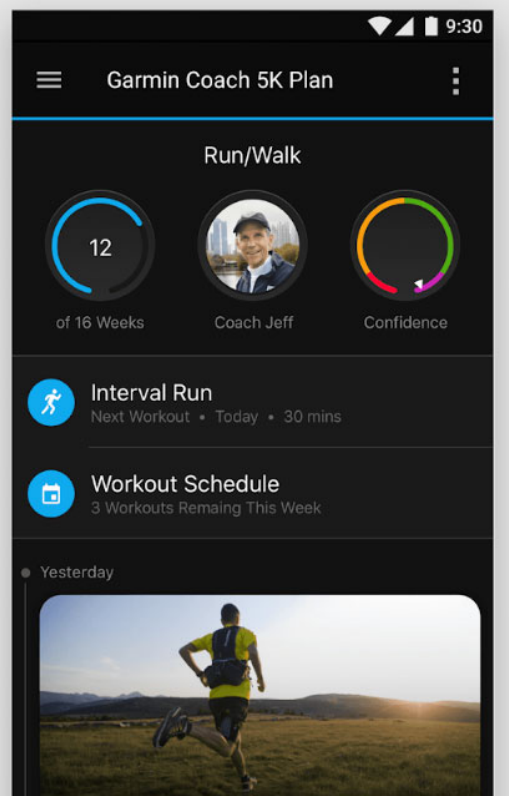

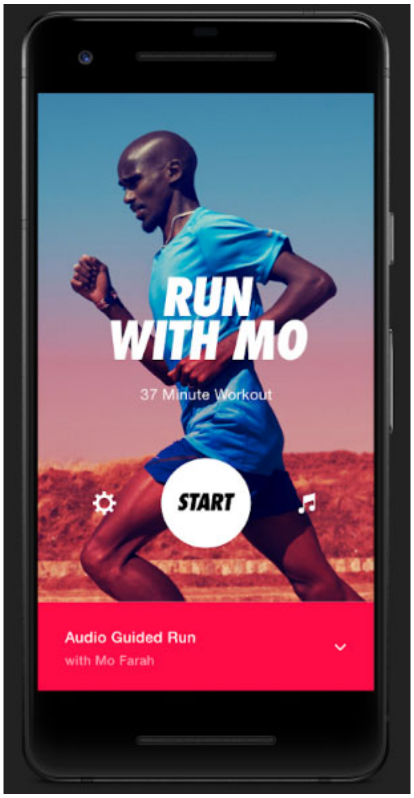

Activity tracking

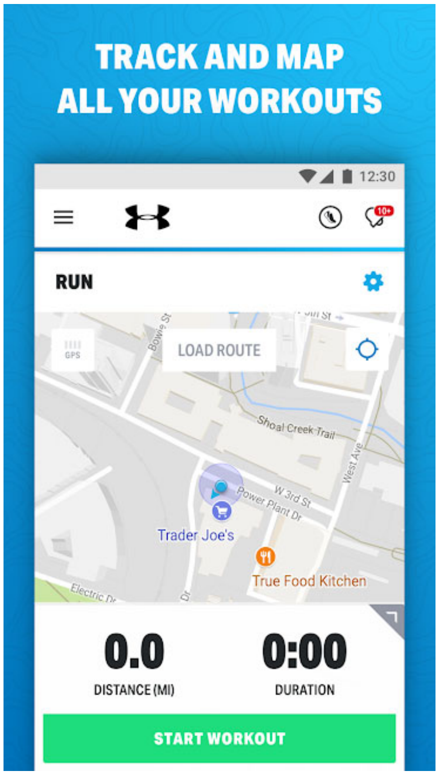

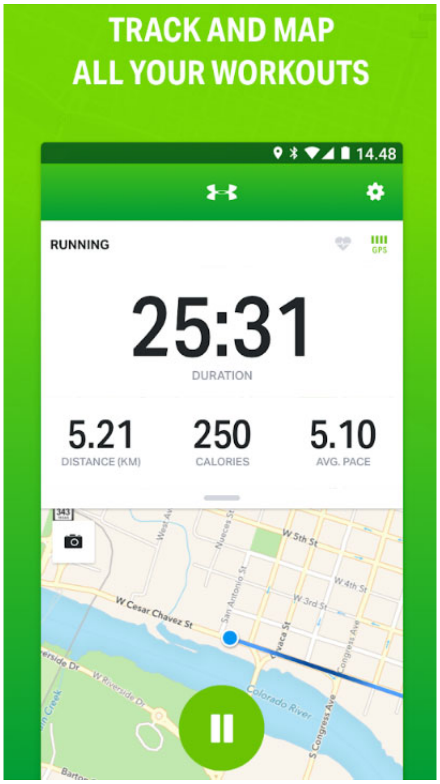

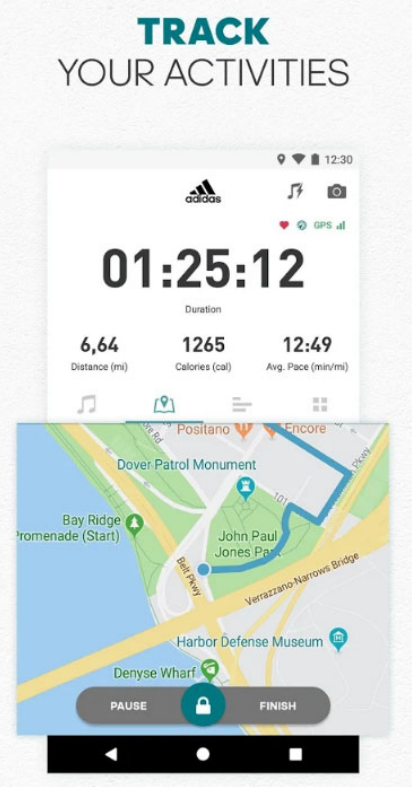

Mood and sleep tracking

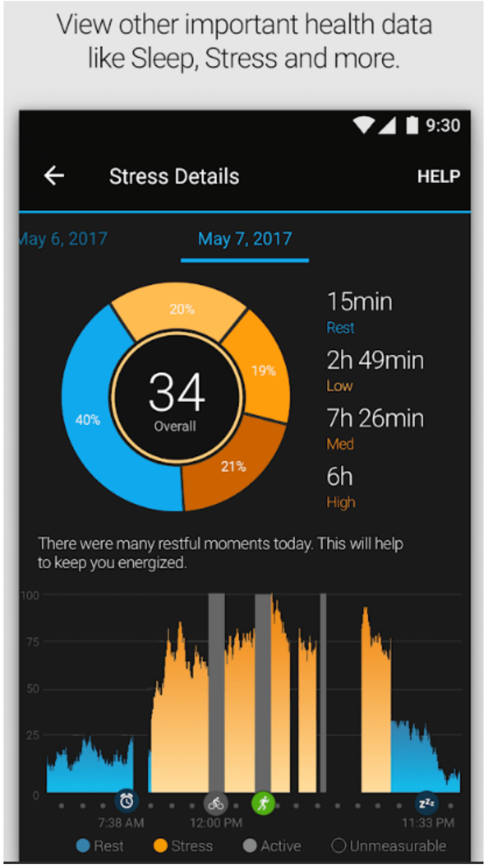

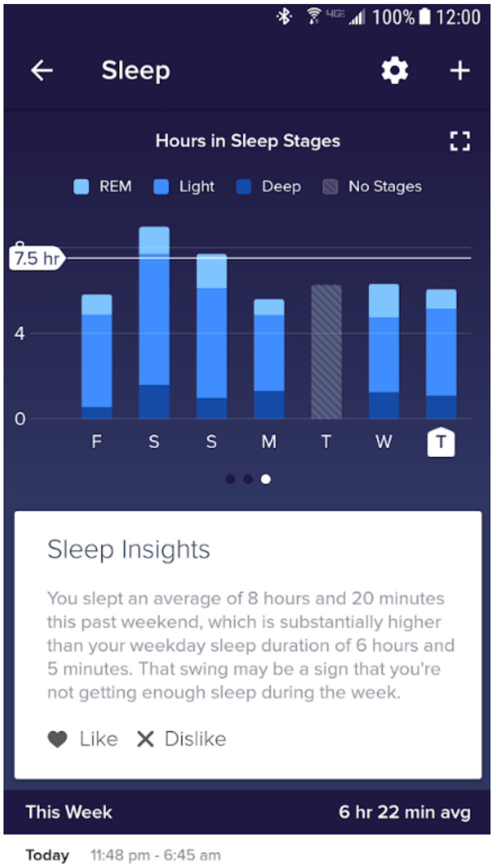

Feedback

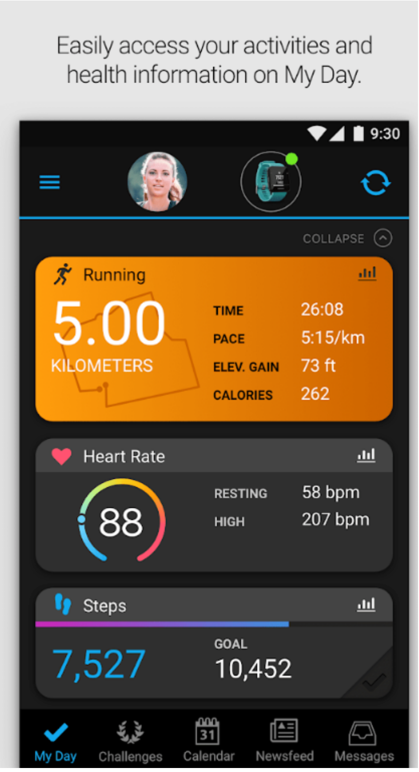

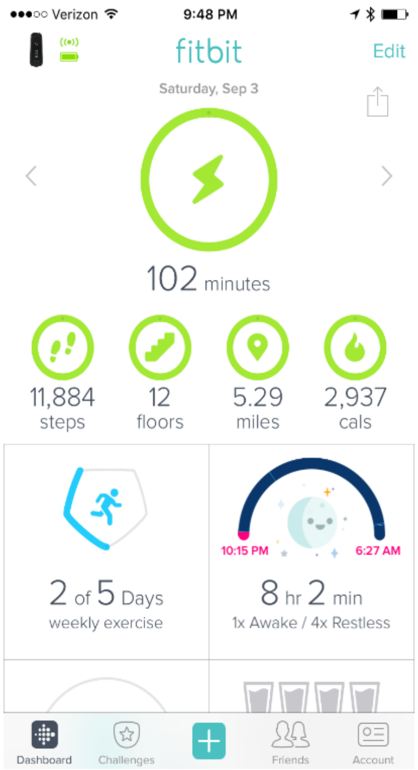

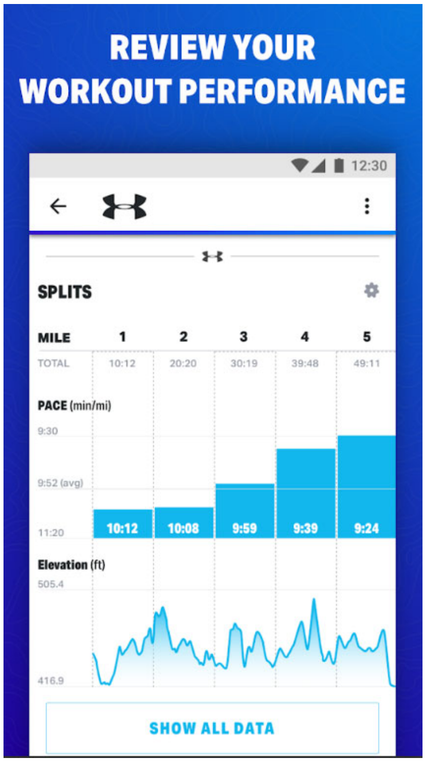

Sharing workout results via social networks

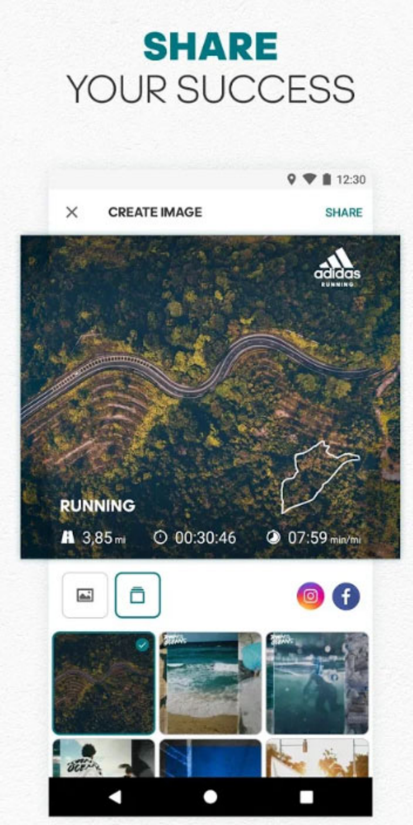

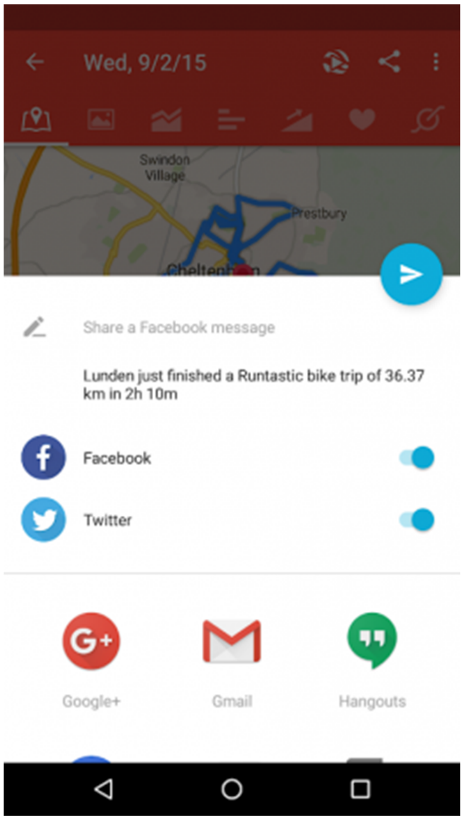

Social support and comparison (in-app social profile and challenges)

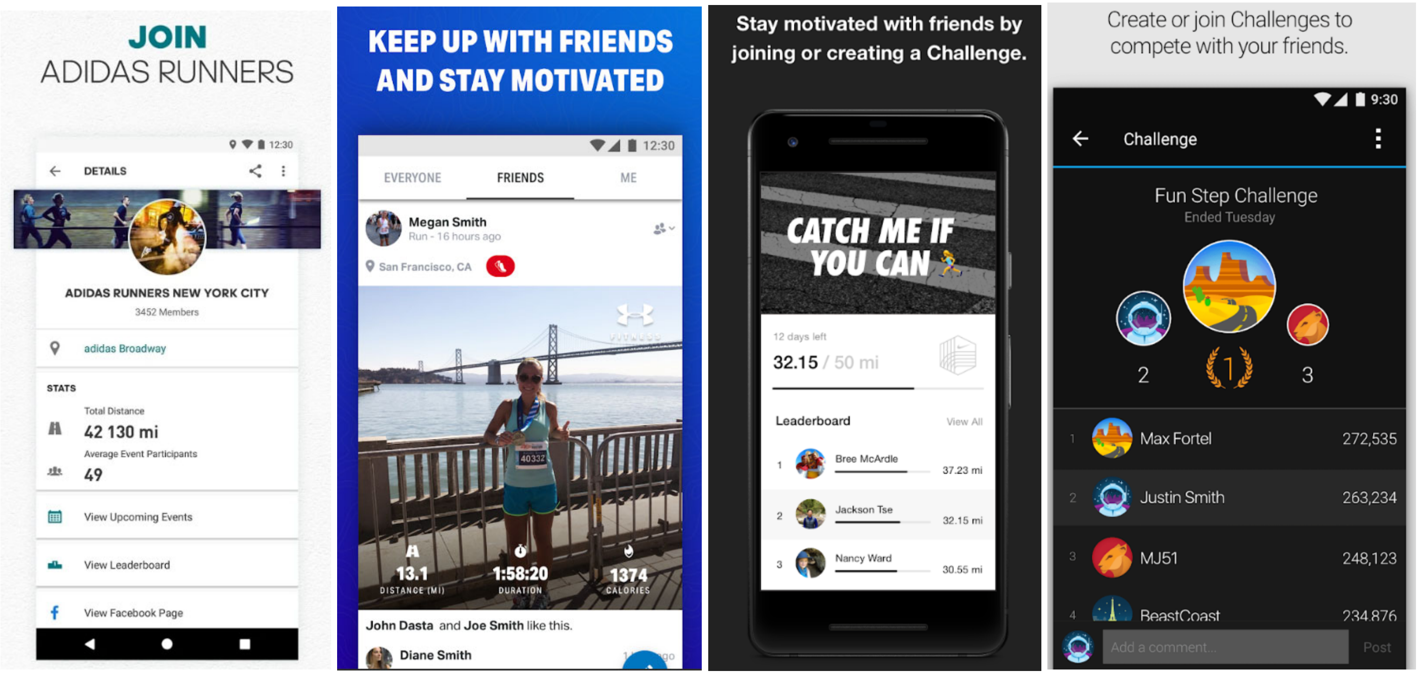

Location tracking

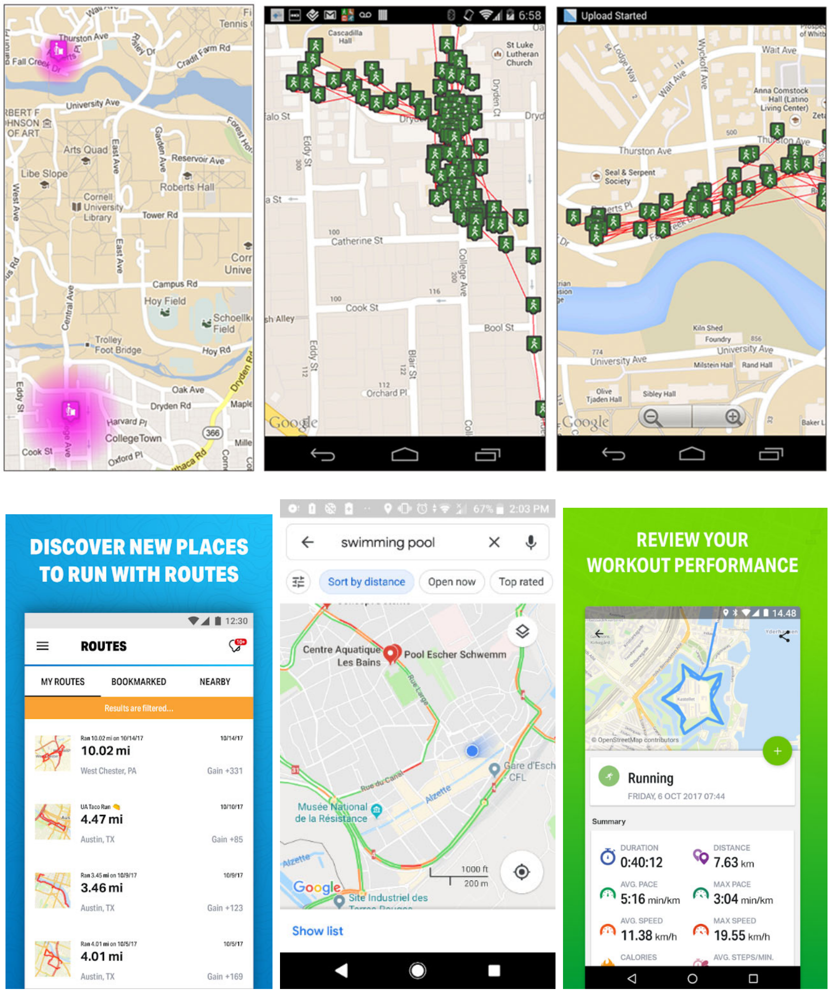

Automatic activity recognition

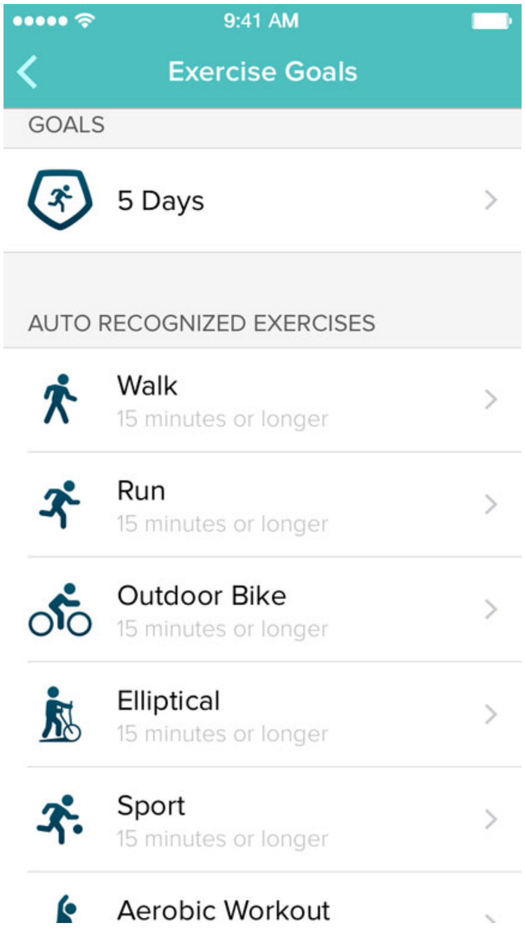

Digital avatar and coach

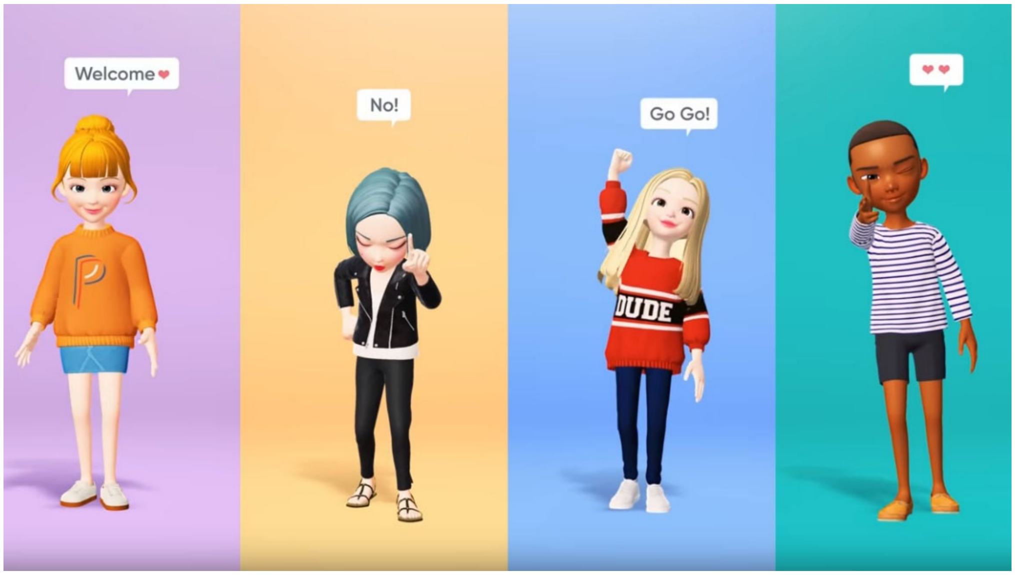

Rewards

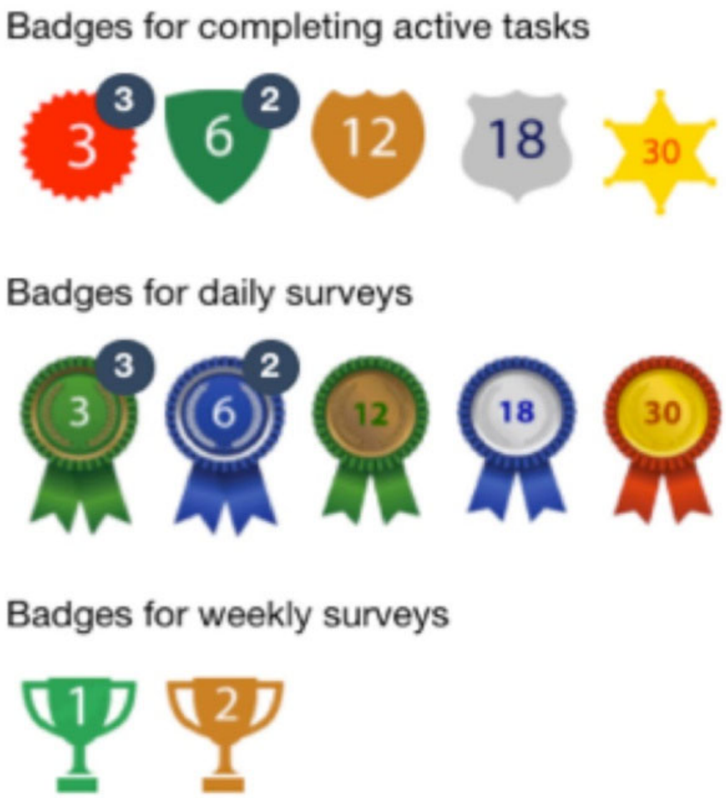
Badges for completing active tasks
3
3
6
2
12
18
30
Badges for daily surveys
3
3
6
2
12
18
30
Badges for weekly surveys
1
2

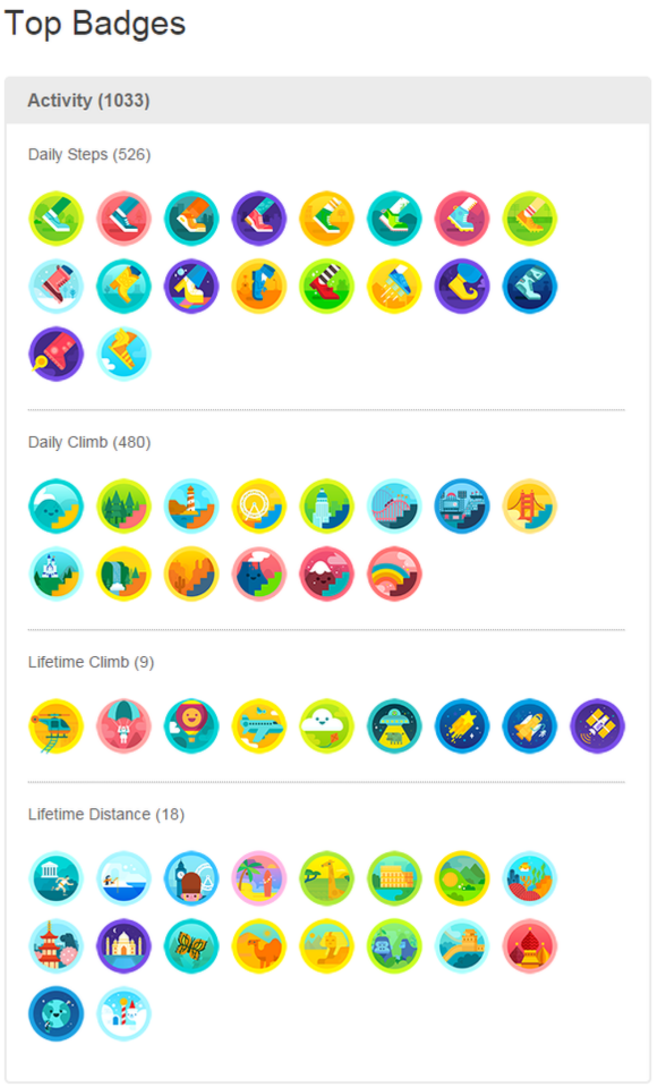
Top Badges
Activity (1033)
Daily Steps (526)
Daily Climb (480)
Lifetime Climb (9)
Lifetime Distance (18)

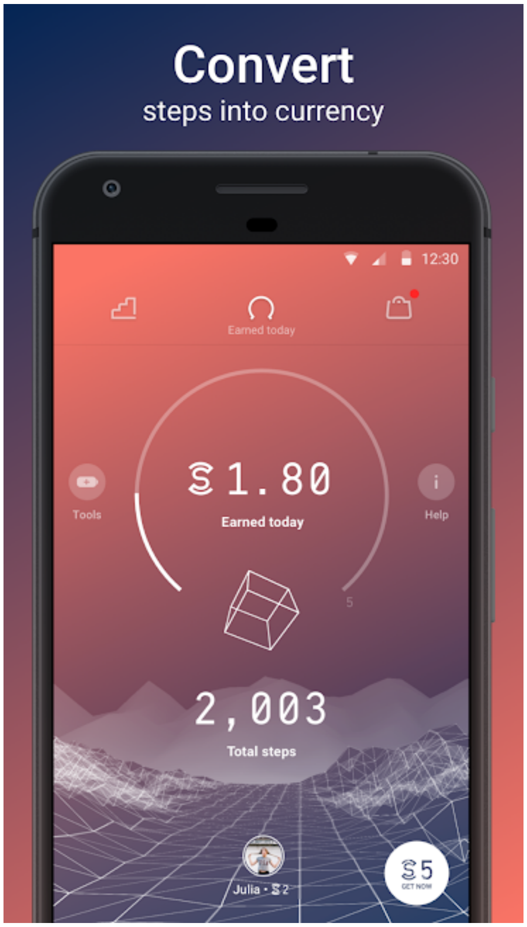
Convert
steps into currency
12:30
Earned today
1.80
Earned today
Tools
Help
2,003
Total steps

Reminders

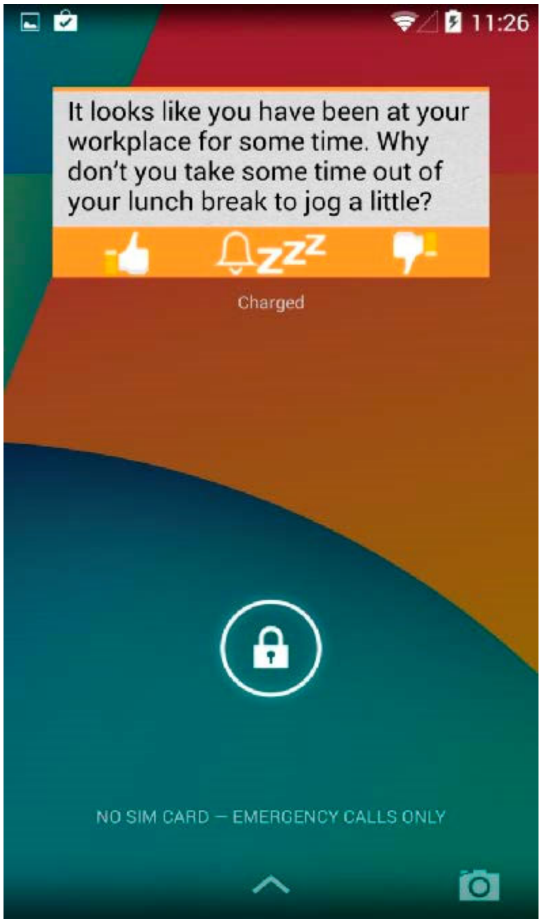
11:26
It looks like you have been at your workplace for some time. Why don't you take some time out of your lunch break to jog a little?
Charged
NO SIM CARD — EMERGENCY CALLS ONLY

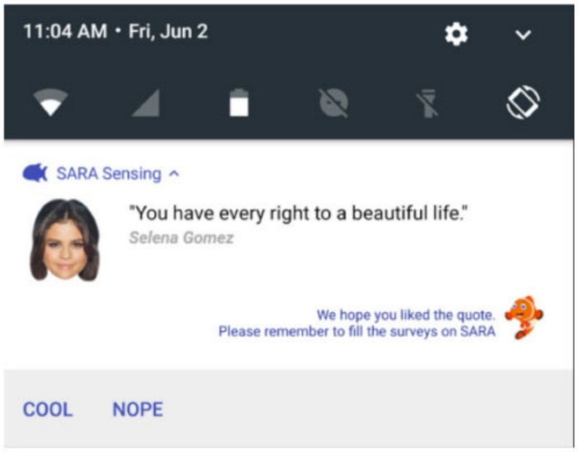
11:04 AM • Fri, Jun 2
SARA Sensing
"You have every right to a beautiful life."
Selena Gomez
We hope you liked the quote.
Please remember to fill the surveys on SARA
COOL
NOPE

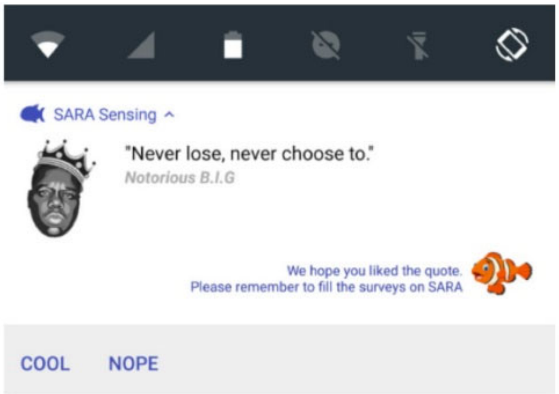
SARA Sensing
"Never lose, never choose to."
Notorious B.I.G
We hope you liked the quote.
Please remember to fill the surveys on SARA
COOL
NOPE
